# Continuous Myoelectric Prediction of Future Ankle Angle and Moment Across Ambulation Conditions and Their Transitions

**DOI:** 10.3389/fnins.2021.709422

**Published:** 2021-08-18

**Authors:** Erika V. Zabre-Gonzalez, Lara Riem, Philip A. Voglewede, Barbara Silver-Thorn, Sara R. Koehler-McNicholas, Scott A. Beardsley

**Affiliations:** ^1^Department of Biomedical Engineering, Marquette University and Medical College of Wisconsin, Milwaukee, WI, United States; ^2^Department of Mechanical Engineering, Marquette University, Milwaukee, WI, United States; ^3^Minneapolis Department of Veterans Affairs Health Care System, Minneapolis, MN, United States; ^4^Department of Rehabilitation Medicine, University of Minnesota, Minneapolis, MN, United States

**Keywords:** autoregressive model (NARX), myoelectric control, gait prediction, level walking, stair ambulation, active powered prosthesis, ankle-foot orthosis and prosthesis

## Abstract

A hallmark of human locomotion is that it continuously adapts to changes in the environment and predictively adjusts to changes in the terrain, both of which are major challenges to lower limb amputees due to the limitations in prostheses and control algorithms. Here, the ability of a single-network nonlinear autoregressive model to continuously predict future ankle kinematics and kinetics simultaneously across ambulation conditions using lower limb surface electromyography (EMG) signals was examined. Ankle plantarflexor and dorsiflexor EMG from ten healthy young adults were mapped to normal ranges of ankle angle and ankle moment during level overground walking, stair ascent, and stair descent, including transitions between terrains (i.e., transitions to/from staircase). Prediction performance was characterized as a function of the time between current EMG/angle/moment inputs and future angle/moment model predictions (prediction interval), the number of past EMG/angle/moment input values over time (sampling window), and the number of units in the network hidden layer that minimized error between experimentally measured values (targets) and model predictions of ankle angle and moment. Ankle angle and moment predictions were robust across ambulation conditions with root mean squared errors less than 1° and 0.04 Nm/kg, respectively, and cross-correlations (R^2^) greater than 0.99 for prediction intervals of 58 ms. Model predictions at critical points of trip-related fall risk fell within the variability of the ankle angle and moment targets (Benjamini-Hochberg adjusted *p* > 0.065). EMG contribution to ankle angle and moment predictions occurred consistently across ambulation conditions and model outputs. EMG signals had the greatest impact on noncyclic regions of gait such as double limb support, transitions between terrains, and around plantarflexion and moment peaks. The use of natural muscle activation patterns to continuously predict variations in normal gait and the model’s predictive capabilities to counteract electromechanical inherent delays suggest that this approach could provide robust and intuitive user-driven real-time control of a wide variety of lower limb robotic devices, including active powered ankle-foot prostheses.

## Introduction

Human locomotion continuously adapts to changes in the environment to maintain balance, reacts to unpredictable perturbations, and predictively adjusts walking patterns to changes in the terrain (Pearson, 2000; Choi and Bastian, 2007). Gait adaptability is a major challenge to lower limb amputees due to the limitations in prostheses and control algorithms (Kannape and Herr, 2016). Accurately and continuously predicting variations in gait, particularly during transitions and noncyclic activities, is limited in commercially available lower limb prostheses. Gait adaptability could be improved by incorporating information on user intent (e.g., myoelectric control), allowing users to modify prosthetic joint dynamics in a more natural, and less physically and cognitively demanding way.

Developing control algorithms for lower limb prostheses is challenging and impacts the level of human adaptation to the device (Huang et al., 2016). The use of finite state machines (FSM) in combination with mechanical intrinsic sensors embedded in the prosthesis itself (Cherelle et al., 2014; Sun et al., 2014; Culver et al., 2018) or worn on the residual limb (Au et al., 2008; Hoover et al., 2013; Kannape and Herr, 2014; Spanias et al., 2018) has become an increasingly common approach due to their high precision and reliability. However, FSM-based control is limited by the relatively small number of pre-defined, discrete states, the need to define switching rules for transitioning between states, and the inability to deal with novel movements.

Alternate approaches have been developed that would continuously determine the dynamic changes of the joint and would not require division into discrete states. Impedance and stiffness control based on joint moment and angle have an increasingly common approach for actuating active powered (i.e., able to generate power during propulsion) lower limb prostheses (Au et al., 2008; Ha et al., 2011; Hoover et al., 2013; Kannape and Herr, 2014; Culver et al., 2018; Klein and Voglewede, 2018; Spanias et al., 2018). Specifically, ankle angle and ankle moment are common targets for controlling transtibial prostheses. The continuous estimation of joint moments has focused on the use of multi-body dynamic musculoskeletal modeling. While effective, it requires constant and time consuming (e.g., 30 min; Meyer et al., 2017) re-calibration of model parameters that are sensitive to changes in muscle-tendon geometry which may not be well characterized for amputees or orthopedic impaired individuals (Shao et al., 2009; Meyer et al., 2017), and consequently, not suitable for real-time applications. Limb joint mechanics and kinematics have been continuously estimated from electromyography (EMG) signals (Sepulveda et al., 1993; Lee and Lee, 2005; Shao et al., 2009; Prasertsakul et al., 2012; Zhang et al., 2012; Chen et al., 2013, 2018; Ardestani et al., 2014; Farmer et al., 2014; Ngeo et al., 2014; Li et al., 2015; Liu et al., 2017a, 2020; Meyer et al., 2017; Huihui et al., 2018; Baby Jephil et al., 2020; Gupta et al., 2020; Keleş and Yucesoy, 2020; Wang et al., 2020), hip joint dynamics (Embry et al., 2018; Dey et al., 2019; Eslamy and Alipour, 2019), knee joint dynamics (Joshi et al., 2011; Embry et al., 2018; Eslamy and Alipour, 2019), force myography (Kumar et al., 2021), and ground reaction forces (GRF) (Liu et al., 2009; Jacobs and Ferris, 2015), among others. Support vector regression (SVR) and Gaussian process regression have been used to continuously estimate ankle angle and ankle moment simultaneously using hip and knee joint kinematics (Dey et al., 2019) and shank kinematics (Eslamy and Alipour, 2019), respectively. Although simultaneous estimation of ankle angle and moment was achieved, performance was characterized during a single type of terrain, i.e., level walking, and implementation would require deducing user intent indirectly from mechanical extrinsic or intrinsic prosthetic sensors.

For a seamless and intuitive device actuation, controllers must recognize the user’s locomotive intention given changes in the environment. Even though mechanical intrinsic sensors have high repeatability and reproducibility, they introduce an intrinsic delay and the resulting control must infer human intention through secondary information such as gait events or joint mechanics. Therefore, their actuation is reactive to user’s biomechanical changes. Surface EMG activity enables a direct prediction of intended biomechanics given that muscle activity precedes force generation, and consequently, limb movement, on the order of 10 ms (Cavanagh and Komi, 1979). Still, the use of EMG sensors poses challenges in achieving robust control due to low signal quality, variability associated with sensor placement and electrode-skin conductivity, cross-talk between nearby muscles, and signal processing for feature extraction.

Classification algorithms have been used together with surface EMG to distinguish among discrete locomotion modes (Huang et al., 2009; Young et al., 2014; Gupta and Agarwal, 2017; Liu et al., 2017b) while other approaches continuously estimate ankle joint kinematics (Sepulveda et al., 1993; Prasertsakul et al., 2012; Zhang et al., 2012; Farmer et al., 2014; Chen et al., 2018; Huihui et al., 2018; Baby Jephil et al., 2020; Gupta et al., 2020; Keleş and Yucesoy, 2020; Wang et al., 2020) and kinetics (Sepulveda et al., 1993; Ardestani et al., 2014; Baby Jephil et al., 2020; Keleş and Yucesoy, 2020) using EMG signals. Most approaches characterize performance during a single type of terrain (e.g., level walking) (Prasertsakul et al., 2012; Zhang et al., 2012; Ardestani et al., 2014; Farmer et al., 2014; Chen et al., 2018; Gupta et al., 2020; Keleş and Yucesoy, 2020; Wang et al., 2020) or ankle motion while sitting (Zhang et al., 2012; Huihui et al., 2018; Baby Jephil et al., 2020). Models that estimate ankle angle or ankle moment during more than one condition (e.g., speeds) have begun to emerge. A deep belief network and principal component analysis, for EMG dimensionality reduction from ten muscle signals, individually combined with a nonlinear back-propagation network have been used to estimate hip, knee, and ankle angle of healthy participants (Chen et al., 2018). Trained generalized inter-subject networks continuously estimated changes in speeds during level walking. Gupta et al. proposed separate subject-specific autoregressive models for five individual terrain types (level walking, stair ascent, stair descent, ramp ascent, ramp descent) to estimate ankle angle using two able-bodied lower limb EMG signals and knee angle (Gupta et al., 2020). A generalized inter-subject wavelet neural network (WNN) and feedforward artificial neural network (FFANN) are capable of estimating ankle moments using EMG activity from eight muscles and two GRFs of patients with unilateral knee replacement while performing three rehabilitation therapy walking programs (Ardestani et al., 2014). Keleş et al. achieved the simultaneous estimation of ankle angle and moment during level walking using a time-delay FFANN and simulated EMG data of a healthy population (Keleş and Yucesoy, 2020). These studies support the feasibility of continuously estimating ankle joint angles and ankle moments independently and simultaneously using EMG signals. However, simultaneous estimation of both ankle angle and moment across multiple types of terrains (e.g., level walking, stair ascent/descent) including transitions between them has not been demonstrated. Moreover, almost all estimations of ankle angle and moment were reactive rather than predictive (i.e., future estimates). A predictive approach would help overcome delays in sensing, processing, and actuation of the mechanical device and also help to modify gait proactively in response to upcoming changes in terrain.

An EMG-driven nonlinear autoregressive neural network with exogenous inputs (NARX) with predictive future states can address these challenges and provide a robust and intuitive control of active powered ankle-foot prostheses. Previous work has demonstrated the ability of a single-output feedforward (open-loop) NARX model to continuously predict future ankle angle of the prosthesis using within-socket EMG activity from the residual limb of transtibial amputees (Silver-Thorn et al., 2012; Farmer et al., 2014). The incorporation of natural, yet abnormal, EMG signals significantly reduced average errors in ankle angle during the gait cycle and phase transitions. However, the subject-specific model was limited to the prediction of a single ambulation condition (level treadmill walking) and did not estimate ankle moments needed in stiffness and impedance control.

In this study, the feedforward NARX model architecture was expanded to a *multiple*-output model that provided simultaneous estimates of future intended state of ankle angle and ankle moment across *multiple* ambulation conditions using lower limb surface EMG signals as input. Ankle plantarflexor and dorsiflexor EMG signals (antagonistic muscles) from healthy young adults were used to continuously predict normal ranges of ankle angle and moment during level overground walking, stair ascent, and stair descent, including transitions between terrains (i.e., transitions to/from staircase). Prediction performance was quantified using novel data sets and characterized as a function of the model parameters (prediction interval, sampling window, and number of hidden units) to identify optimal subject-specific parameters that minimized error. Models were trained and optimized for each participant to account for individual’s specific variations of EMG activity and limb dynamics. The suitability of the model prediction for prosthetic control was then examined by statistically analyzing the prediction variability at critical performance points (Protopapadaki et al., 2007; Sinitski et al., 2012; Loverro et al., 2013) within ambulation conditions where excessive deviations could lead to trips or falls. Gait intention, via lower limb EMG signals, was explored by quantifying the impact of EMG inputs on the model prediction of ankle angle and moment.

## Materials and Methods

### Participants

Ten healthy young adults (7 males; age = 21.9 ± 1.4; mass = 72.5 ± 8.8 kg; height = 1.8 ± 0.09 m) participated in the study. Participants were excluded if they presented neurologic or orthopedic impairments that would affect their ability to walk or follow instructions. The study was approved by the Institutional Review Board at Marquette University (Milwaukee, Wisconsin), and all participants provided written informed consent.

### Experimental Procedure

**FIGURE 1:**
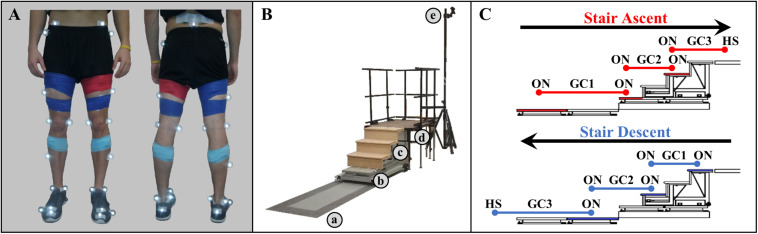
**(A)** Modified Helen Hayes infrared lower limb marker set and EMG sensor placement. **(B)** Experimental walkway setup including (a) force plates embedded in the floor, (b) stair force plates, (c) staircase, (d) landing platform, and (e) infrared motion cameras. **(C)** Schematic of step-over-step stair ambulation gait cycles (GC). Foot contact occurred on the shaded force plates (red, stair ascent; blue, stair descent).

During a single experimental session, participants ambulated at a self-selected speed wearing athletic shoes in three different ambulation conditions, level overground walking (LW), stair ascent (AS), and stair descent (DS). Twenty-five reflective markers were placed on the participant’s key anatomical landmarks (posterior superior iliac spine and bilaterally on the anterior superior iliac spine, greater trochanters, thighs, medial and lateral femoral condyles, shanks, medial and lateral malleoli, calcaneus, second and fifth metatarsal heads, anterior end of first distal phalanx) to define seven lower body segments (pelvis, thighs, shanks, feet) based on a modified Helen Hayes marker set (Figure 1A). Trigno^TM^ wireless surface EMG electrodes (Delsys, Inc., Natick, MA, United States) were placed bilaterally over the tibialis anterior (dorsiflexor), and the gastrocnemius medialis (plantarflexor). Anthropometric measures (height and weight) were then taken. The walkway was instrumented with two 3-dimensional 6-channel force plates (Advanced Mechanical Technology, Inc., Watertown, MA, United States) embedded in the floor, a modified 4-step (17.78 cm rise, 60.45 cm width, 29.10 cm run; 1st step: 46.34 cm width, 26.45 cm run) instrumented staircase (Advanced Mechanical Technology, Inc., Watertown, MA, United States) and a landing platform (1.22 m × 0.91 m) (Figure 1B). To minimize session duration and set-up time, ambulation conditions were not randomized. Prior to data collection, participants walked on the walkway to get accustomed to the researcher instructions and staircase setup. First, during stair ascent trials, participants traversed the walkway (∼3 m), ascended the stairs in a step-over-step fashion, and walked to the end of the landing platform (AS trial). Each stair ascent trial was followed by a subsequent stair descent trial, during which participants turned when instructed, crossed the platform, descended the stairs, step-over-step, and returned to their starting position (DS trial). For level ground walking, the staircase and landing platform were removed and participants walked the entire length of the walkway (∼5 m). Participants were encouraged to take breaks as needed to minimize potential fatigue. A minimum of 15 trials were completed for each ambulation condition.

### Data Acquisition and Signal Processing

Surface EMG activity, kinetic, and kinematic data were collected and synchronized. Surface differential EMG recordings were amplified (909 V/V), sampled at 1,200 Hz, filtered to obtain linear envelopes, and down sampled to 120 Hz. EMG linear envelopes were obtained using a band-pass filter from 20 to 499.5 Hz (4th order zero-phase Butterworth), followed by full-wave rectification, and a low-pass filter with a 5.5 Hz cutoff frequency (4th order zero-phase Butterworth). Kinetic data were sampled at 1,200 Hz, low-pass and notch filtered (4th order zero-phase Butterworth) at 15 Hz and 59–61 Hz, respectively, and down sampled to 120 Hz. Kinematic data were sampled at 120 Hz using an OptiTrack (NaturalPoint, Inc., Corvallis, OR, United States) motion capture system (14 to 16 Flex 13 cameras). Markers were manually identified using AMASS (C-Motion, Inc., Germantown, MD, United States) software and processed in Visual 3D (C-Motion, Inc., Germantown, MD, United States) to extract limb kinematic (ankle angle) and foot kinetic (ankle moment, normalized to participant’s body mass) time series and gait events. Marker trajectories, and kinematic and kinetic time series were subsequently low-pass filtered (15 Hz, 4th order zero-phase Butterworth) and interpolated (3rd order polynomial, max. gap of 20 frames) in Visual 3D. Ankle angle in the sagittal plane was computed as the motion of the foot segment relative to the shank segment coordinate system using Euler angles. Ankle moment in the sagittal plane was calculated using conventional inverse dynamics and resolved to the shank segment coordinate system (C-motion, 2015).

Gait events were defined kinematically as HS for heel strike and TO for toe off on floor, and kinetically as ON for first foot contact on force plate, OFF for last foot contact on force plate (threshold 10 N). All trials were temporally normalized and truncated from 225 ms before the first heel strike on the first force plate to the first heel strike before contralateral toe off on the last force plate (percent trial). As a result, level walking condition consisted of one gait cycle and each stair ambulation condition consisted of three continuous gait cycles, as of traversing from level walking to stair stepping to level waking (Figure 1C). Staircase transitions, the short instance when transitioning between terrains, were defined from the start of the swing phase of the limb being investigated to the start of the swing phase of the contralateral limb of the transition step, except during the transition from the platform to the staircase (stair descent) where the transition limb was the contralateral limb.

### NARX Neural Network Model

**FIGURE 2:**
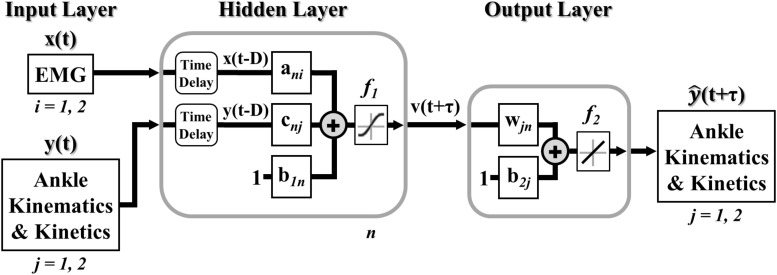
Multiple-input multiple-output feedforward (open-loop) NARX model. EMG linear envelopes (ankle dorsiflexor and plantarflexor) and experimentally measured values of ankle angle and ankle moment were weighted and fed via tapped delay lines to a single hidden layer containing nonlinear units with hyperbolic tangent sigmoid transfer functions. Intermediate outputs were weighted and linearly combined to provide continuous and simultaneous predictions of future ankle angle and moment over time.

A model of lower limb state was developed to continuously predict simultaneous ankle kinematics and kinetics across ambulation conditions and terrain transitions. Specifically, a feedforward (open-loop) multiple-input multiple-output NARX model (Leontaritis and Billings, 1985; Narendra and Parthasarathy, 1990) was created, trained, and tested in MATLAB (R2017a, The MathWorks Inc., Natick, MA, United States) using the Neural Network Toolbox. The feedforward NARX model consisted of an input layer containing the windowed EMG linear envelopes of the ankle dorsiflexor and plantarflexor and the experimentally measured values of ankle angle and ankle moment (targets) passed through separate tapped delay lines, a single hidden layer containing nonlinear units, and a linear output layer containing separate outputs for the predicted (i.e., future estimates) ankle angle and moment in the sagittal plane (Figure 2).

The feedforward NARX model output, ${\hat{y}}_{j}$*(t+m)*, at each time point was calculated as,

(1) $$v_{n}\left(t+m\right)=f_{1}(\sum_{q=0}^{d}\sum_{i=1}^{2}a_{n\,i}\left(q\right)x_{i}\left(t-q\right)-\sum_{q=1}^{d}\sum_{j=1}^{2}c_{n\,j}\left(q\right)y_{j}\left(t-q\right)+b_{1}n),n=1,2,\ldots ,N$$

(2) $${\hat{y}}_{j}\,\left(t+m\right)=f_{2}\,\left(\sum_{n=1}^{N}w_{j\,n}\,v_{n}\,\left(t+m\right)+b_{2\,j}\right),j=1,2$$

where *v_*n*_(t+m)* was the output of nth unit in the hidden layer, *N* was the total number of hidden units, *m* was the prediction interval in time steps (τ = *mΔt*), *d* was the sampling window length (*D = dΔt*), *x_*i*_(t-q)* was the input of the ith EMG linear envelope for the prior *q* time step, *y_*j*_(t-q)* was the past jth desired target value (ankle angle and moment), *a*_*ni*_, *c*_*nj*_, and *w*_*jn*_ were the weights of EMG inputs, desired target values, and ankle angle and moment outputs, respectively, *b*_1n_ and *b*_2j_ were the bias weights at the hidden and output layers, respectively, *f*_1_ was a nonlinear hyperbolic tangent sigmoid function, and *f*_2_ was a linear function with unit slope. The sampling window specified the number of prior input values over time (exogenous and targets) used to calculate future ankle angle and moment. The prediction interval specified the time between the current inputs (exogenous and targets) and the model output predictions of future ankle angle and moment.

Separate models were trained for each participant. During training, ten randomized trials from each ambulation condition of a single limb were organized as a concurrent set of sequences and divided into contiguous blocks where 80-percent (8 complete trials/condition) were used for training and 20-percent (2 complete trials/condition) were used for validation. An additional trial of each ambulation condition (novel test trial) was held back and used to separately assess model performance after training using a leave-one-out 10-fold cross-validation. Each model was trained and optimized to minimize the mean squared error (MSE) between the ankle angle and moment targets and the model predictions using a Levenberg-Marquardt backpropagation supervised learning procedure. To fit ankle angle and moment equally, training errors (i.e., MSE) were normalized to the range of [−2, 2] corresponding to normalizing model predictions and targets between −1 and 1 using a min-max mapping of the k-fold training dataset. For each training dataset, ten networks were trained using different initial weights and biases to improve shallow network generalization and avoid overfitting. The network with the lowest MSE averaged across ambulation conditions was selected as the generalized network for that k-fold dataset. To explore the capabilities of the network, NARX model performance was characterized as a function of the prediction interval (τ; 33, 42, 50, 58, 67, 75, 83, 108, 142 ms), sampling window (D; 8, 17, 33, 50, 67, 83 ms), and number of hidden units (N; 2 to 16 in steps of 2) with error goal bounded to 1-percent of the moment variance of all recorded trials (1% training error goal). Subsequently, while the prediction interval was fixed to 58 ms (7 time steps) to counteract electromechanical inherent delays of the Marquette University’s ankle-foot prosthesis (Sun and Voglewede, 2012; Sun et al., 2014; Klein and Voglewede, 2018), minimum MSE averaged over all novel test trials and ambulation conditions (10 complete trials/condition) was used to determine the optimal sampling window and number of hidden units. The training process was then repeated using the fixed prediction interval and the optimal sampling window and number of hidden units with an error goal of zero to maximize network performance for each participant. This optimized subject-specific network structure was used to evaluate model performance after training, unless otherwise specified.

### Model Performance Measurements and Statistical Analysis

The number of participants included in the analysis of stair ambulation conditions was reduced to eight because two participants initiated trials with the limb opposite to the one being analyzed. All performance measurements and statistical analysis were averaged across ten novel test trials and then across participants for each ambulation condition (LW, *n* = 10; AS and DS, *n* = 8).

Root mean square error (RMSE) and coefficient of determination (R^2^) were calculated between the target and model prediction of ankle angle and moment for each test trial to evaluate model performance. The coefficient of determination (R^2^), obtained from squaring the cross-correlation peak, was used to quantify the ability of the model to reproduce the temporal profiles of angle and moment for each ambulation condition and their transitions.

Using the first set of models (1% training error goal), simple linear regressions were performed to examine the effects of prediction interval, sampling window, and number of hidden units on model performance. For each model output and ambulation condition, a linear fit (slope and intercept) was performed in MATLAB (R2017a) using the average RMSE collapsed along a single model parameter dimension (i.e., RMSE averaged across two of the three model parameters). Goodness of fit was assessed by the coefficient of determination (R^2^), and an ANOVA (*p* < 0.001) was performed for each model parameter and ambulation condition to determine whether the fitted slope was significantly different from zero.

Using the second set of models (optimized subject-specific network structure), RMSE was computed over each trial to characterize maximal model performance across participants. To evaluate the impact of EMG signals on model performance, the instantaneous RMSE over time of NARX model predictions (time-varying EMG) were compared against errors of NARX models having constant EMG inputs. Constant EMG inputs, *x* = $\bar{x}$, for each participant, were calculated as the average EMG signal over time for each test trial and ambulation condition to provide the same average signal power while omitting the time-varying information. To facilitate analysis across test trials and participants of instantaneous RMSE, individual trials were interpolated to a common length for each ambulation condition (LW: 145, AS: 430, DS: 400 samples).

Two types of critical performance points, clearance intervals (Loverro et al., 2013) and stance critical points (Protopapadaki et al., 2007; Sinitski et al., 2012), were assessed for each ambulation condition to verify that the NARX model predictions where within the variability of the measured targets. Staircase leg dynamics in this study was matched to the steps from Loverro’s et al. 7-step staircase, and clearance intervals were selected corresponding to the locations of minimum foot and toe clearance, i.e., points with the highest tripping risk. Intervals were defined by the range of timings (mean ± standard deviation, i.e., 30 total points) of the minimum clearance angle. Single stance critical points (i.e., nineteen points) were extracted at crucial kinematic (TO, maximum dorsiflexion, maximum plantarflexion) and kinetic events (maximum plantarflexion moment) for prosthetic design. For each critical point, samples were tested for normality using the Shapiro-Wilk test. For normally distributed samples, a paired-samples *t*-test was used to determine if inter-subject NARX predictions were statistically different from that target. Sign test was performed for non-normally and asymmetric distributed critical points. Statistical analyses were performed using SPSS 22 (SPSS Inc., Chicago, IL, United States) with a significance level of *p* < 0.05. The Benjamini-Hochberg (B-H) procedure was used to adjust *p* values with a false discovery rate of 0.05 to correct for multiple comparisons (Benjamini and Hochberg, 1995).

## Results

**FIGURE 3:**
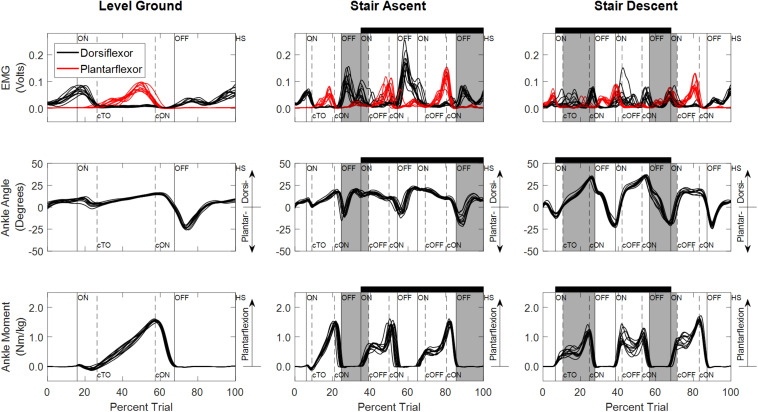
Experimentally measured linear envelope of EMG signals, ankle angle, and ankle moment for level overground walking, stair ascent, and stair descent of a typical participant (S04). Percent trial is normalized from 225 ms before first heel strike on the first force plate to the first heel strike before contralateral toe off of the last force plate. Vertical lines denote gait events (solid: limb used to train the model; dashed: contralateral limb) defined based on force plate and floor contact (ON, first contact on force plate; OFF, last contact on force plate; HS, heel strike on floor; TO, toe off on floor). Contralateral gait events are identified by a lowercase “c” (e.g., cTO, contralateral toe off). Staircase ambulation (black horizontal bar) is defined as the first foot contact on the staircase to the first foot contact on level ground of the limb used during training. Staircase transitions to/from level ground are shaded gray. Double limb support occurs when both feet are in contact with the ground simultaneously (ON to cTO *or* ON to cOFF and cON to OFF).

Experimentally measured ankle dorsiflexor and plantarflexor EMGs, ankle angles and ankle moments used to train and test the NARX models of a typical participant (S04) are illustrated in Figure 3. EMG activity was variable across trials, occasionally exhibiting co-activation; however, activation patterns were consistent with the reported literature for all ambulation conditions for all participants (Selk Ghafari et al., 2009; Benedetti et al., 2012; Han et al., 2015). Ankle angle was consistent across trials with the greatest ankle range of motion occurring for stair descent, while ankle moment exhibited more variability.

**FIGURE 4:**
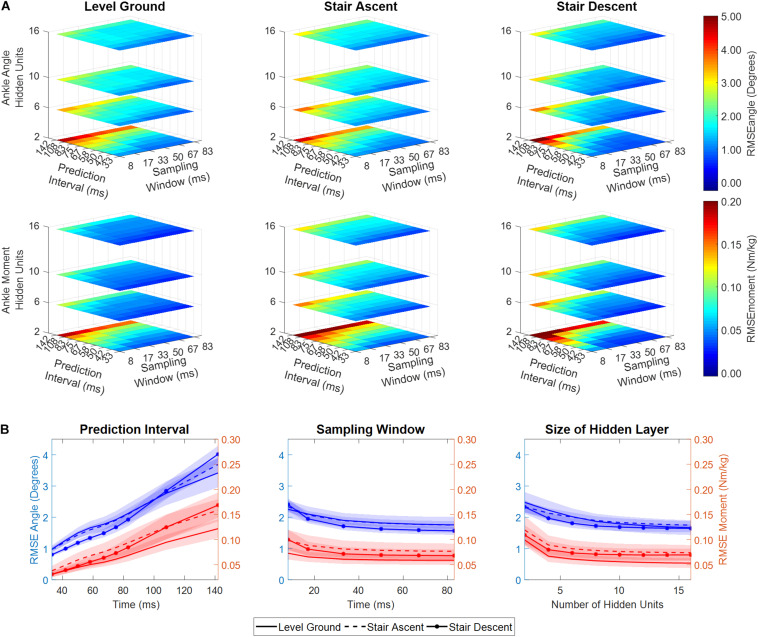
**(A)** RSME between predicted and experimentally measured ankle angle and ankle moment as a function of NARX model prediction interval, sampling window, and number of hidden units, averaged across participants. **(B)** RMSE ankle angle and moment collapsed across model parameters (i.e., averaged across two of the three dimensions). Shaded regions denote ± 1 standard deviation. RMSE is shown for the first set of NARX models trained with error goal bounded to 1-percent of the moment variance of all recorded trials.

The performance of the NARX model was comparable across a wide range of sampling windows, and the number of hidden units; however, it was dependent on the size of the prediction interval (Figure 4). As prediction interval increased, RMSE had a significant linear increase for ankle angle and moment across ambulation conditions (ANOVA *p* < < < 0.001, R^2^ > 0.98). Model error for predicting ankle angle and moment was largely unaffected by the size of the sampling window across ambulation conditions (ANOVA *p* > 0.009, R^2^ = [0.65, 0.85]) with error saturating after 33 ms (ANOVA *p* > 0.033, R^2^ = [0.81, 0.94]). The number of hidden units used in the network showed a small negative correlation with ankle angle and moment RMSE across ambulation conditions (ANOVA *p* > 0.008, R^2^ = [0.54, 0.71]) that was significant for ankle angle RMSE during level walking and stair ascent (ANOVA *p* < 0.001, R^2^ = 0.88). After 8 hidden units, RMSE saturated for ankle angle and moment across ambulation conditions (ANOVA *p* > 0.003, R^2^ = [0.61, 0.96]). There were minimal differences in angle and moment error among ambulation conditions (Figure 4B). When collapsed across model parameters, RMSE angle, averaged across participants and ambulation conditions, ranged from 0.73° to 1.16° for a 33 ms prediction interval, 1.18° to 2.01° for a 58 ms prediction interval and 2.60° to 4.49° for a 142 ms prediction interval with 1% training error goal. Similarly, RMSE moment ranged from 0.025 to 0.052 Nm/kg, 0.040 to 0.094 Nm/kg, and 0.099 to 0.215 Nm/kg, for 33, 58, and 142 ms prediction intervals, respectively.

**FIGURE 5:**
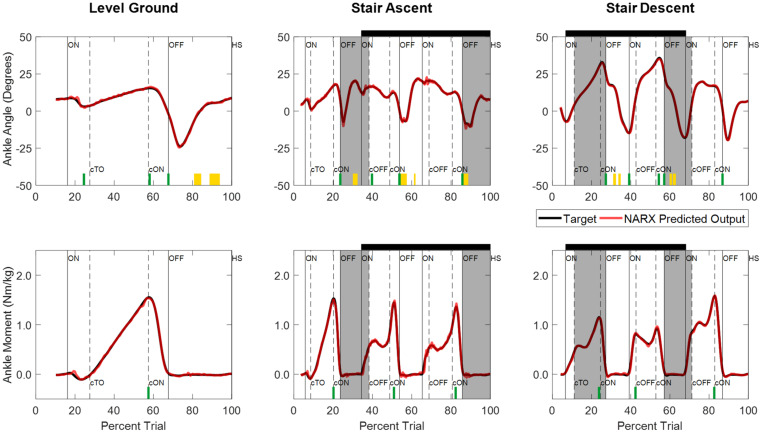
Time series of NARX model prediction of ankle angle and ankle moment for level ground walking, and stair ambulation of a typical participant (S04) using optimal model parameters (τ: 58 ms, D: 83 ms, N: 6). NARX model predictions are shown for the k-fold novel test trials with the best accuracy across ambulation conditions and model outputs. Critical performance points used to test for statistically significant differences between the model prediction and experimentally measured targets are denoted by yellow blocks (clearance intervals), and green lines (stance points). No significant differences were found across participants (B-H adjusted *p* > 0.05). Shading and line markers are defined the same as in Figure 3.

Joint ankle angle and ankle moment model predictions closely matched the experimentally measured targets in all ambulation conditions and staircase transitions as shown in Figure 5. The figure shows the comparison of model predictions and targets of a typical participant using optimal model parameters (τ: 58 ms, D: 83 ms, N: 6). Table 1 lists the mean and standard deviations of the correlations (R^2^) and errors (RMSE) of the NARX model prediction of ankle angle and moment across participants for all ambulation conditions. The results show high levels of accuracy in all ambulation conditions and model outputs. R^2^ ranged between 0.989 and 0.999. All peak cross-correlations occurred at zero time lag. Stair descent had the lowest RMSE and the highest correlations for both ankle angle (RMSE = 0.55 ± 0.13°, R^2^ = 0.999 ± 0.001) and moment (RMSE = 0.025 ± 0.007 Nm/kg, R^2^ = 0.999 ± 0.001). The maximum error occurred in the prediction of ankle angle during level ground walking (RMSE = 0.84 ± 0.23°, R^2^ = 0.989 ± 0.005) and in the prediction of ankle moment during stair ascent (RMSE = 0.036 ± 0.009 Nm/kg, R^2^ = 0.997 ± 0.001). Using the Benjamini-Hochberg multiple comparisons procedure, no significant difference across participants was found between targets and NARX model predictions in any of the critical performance points (B-H adjusted *p* > 0.065). Detailed statistical scores, and mean and standard deviation of ankle angle and moment predictions and targets of all critical points are listed in Supplementary Table 1.

**TABLE 1:** RMSE and R^2^ values of NARX model predictions for each ambulation condition averaged across participants.

Ambulation condition	Model output	$\bar{\text{RMSE}}$	σ_RMSE_	Units	$\bar{{\text{R}}^{2}}$	$\sigma_{R^{2}}$
**Level ground (*n* = 10)**	Angle	0.84	0.23	Degrees	0.989	0.005
	Moment	0.026	0.006	Nm/kg	0.998	0.001
**Stair ascent (*n* = 8)**	Angle	0.78	0.14	Degrees	0.995	0.002
	Moment	0.036	0.009	Nm/kg	0.997	0.001
**Stair descent (*n* = 8)**	Angle	0.55	0.13	Degrees	0.999	0.001
	Moment	0.025	0.007	Nm/kg	0.999	0.001

**FIGURE 6:**
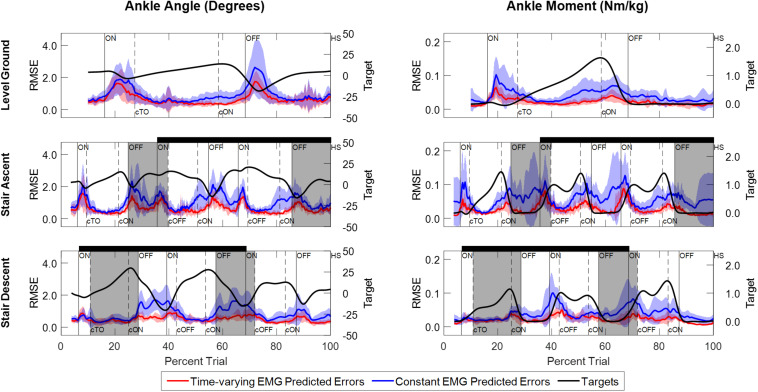
Instantaneous RMSE between experimentally measured values and NARX model predictions of ankle angle and ankle moment across trials and participants. Errors are shown for model predictions using the participant’s time-varying EMG as input (red trace) and for model predictions using the average EMG signal as a constant input (blue trace). Shaded regions denote ± 1 standard deviation. The ankle angle and moment targets averaged across trials and participants are shown for reference (black trace) and scaled accordingly to the right-side vertical axis. Double limb support intervals and gait event markers are defined the same as Figure 3.

Comparison of the instantaneous RMSE over time for the NARX models using the participant’s time-varying EMG as inputs against models using average EMG showed larger errors and increased variability for the constant-EMG NARX predictions across all ambulation conditions and staircase transitions for both ankle angle and ankle moment (Figure 6 and Table 2). The patterns of EMG contribution were consistent among ambulation conditions and model outputs. Removal of the time-varying EMG input had the largest impact during double limb support for both ankle angle and moment. Additionally, the error increased around maximum plantarflexion and going into maximum plantarflexion moment for constant-EMG predictions. The use of time-varying EMG inputs decreased peak errors of ankle angle and moment by approximately 50 and 60% to values less than 1.11° and 0.077 Nm/kg, respectively, across ambulation conditions.

**TABLE 2:** Temporal average of the instantaneous RMSE calculated across participants for the NARX models using the participant’s time-varying EMG as input and the NARX models using the average EMG as a constant input.

Ambulation condition	Model output	Units	Time-varying EMG		Constant EMG	
				
			$\bar{\text{RMSE}}$	σ_RMSE_	$\bar{\text{RMSE}}$	σ_RMSE_
**Level ground (*n* = 10)**	Angle	Degrees	0.67	0.35	0.92	0.51
	Moment	Nm/kg	0.022	0.010	0.039	0.017
**Stair ascent (*n* = 8)**	Angle	Degrees	0.64	0.29	1.02	0.44
	Moment	Nm/kg	0.028	0.015	0.058	0.022
**Stair descent (*n* = 8)**	Angle	Degrees	0.50	0.17	0.82	0.40
	Moment	Nm/kg	0.023	0.009	0.039	0.018

## Discussion

An approach was presented for continuous predictive mapping of lower limb state that incorporated user intent, vis-à-vis surface EMG of the lower limb, to predict future ankle joint kinematics and kinetics simultaneously across ambulation conditions, including transitions between terrains. The single-network, feedforward NARX model had the ability to characterize normal gait patterns of ankle angle and ankle moment with predictions that fell within the experimentally measured variability of the kinematic and kinetic targets across trials and participants.

The autoregressive model presented here continuously models the nonlinear dynamic relationships between muscle activation and ankle dynamics to predict ankle kinematics and kinetics across ambulation conditions and terrain transitions. In contrast, EMG-driven FSMs to control active powered lower limb prostheses typically allow the amputee to select a discrete locomotion mode (Liu et al., 2017b; Spanias et al., 2018) or to control a single parameter (e.g., motor toque gain) during a discrete period of the gait cycle (Au et al., 2008; Kannape and Herr, 2014), and consequently, limit the amputee’s control over the prosthesis. Despite proportional myoelectric approaches that enable continuous prosthetic control throughout the gait cycle using volitional muscle contractions, the volitional actuation of the prosthesis can become physically and cognitively demanding over time (Huang et al., 2016). Here, the NARX model leverages the user’s natural muscle activation patterns to reduce muscle fatigue and the cognitive demand on the user to provide a continuous predictive characterization of gait over time without the need for explicit identification of gait events or selection of ambulation modes. Moreover, the training and optimization of the network structure to maximize individual performance is relevant for the use in prosthetic applications where amputees may develop abnormal muscle activity and gait patterns to maintain stability and compensate for limitations in their prosthesis (Herr and Grabowski, 2012; Huang and Ferris, 2012; Seyedali et al., 2012; Silver-Thorn et al., 2012).

The autoregressive model structure exploits the cyclic process of lower limb motion, to anticipate repetitive components of movement, resulting in a high overall performance (R^2^ > 0.989) while reducing the model degrees of freedom needed to predict limb kinematics and kinetics during gait. The use of time-varying EMG signals during gait resulted in less error compared to model predictions without time-varying information (Table 2 and Figure 6). EMG signals provided an important source of information about limb state that was used to differentiate the temporal profiles of ankle dynamics. Although EMG signals contributed to the model prediction of ankle angle and ankle moment across ambulation conditions, the cyclic nature of walking and the open-loop structure of the feedforward model (which used experimentally error-free ankle angle and moment past values) limited the strength of the EMG contribution to the model predictions across ambulation conditions in healthy young adults. Similar to Farmer et al., while using residual within-socket EMG of transtibial amputees (Farmer et al., 2014), EMG signals had the greatest impact on error in regions where the gait profile was noncyclic, such as transitions to and from single limb support, staircase transitions, and around plantarflexion and moment peaks. Contrary to previous studies in transtibial amputees (Farmer et al., 2014) and able-bodied participants (Gupta et al., 2020), ankle angle accuracy and overall level of EMG contribution did not depend on the range of motion of the ankle, yielding similar levels of error across ambulation conditions and model types tested. These results suggest that EMG signals from the lower leg (ankle dorsiflexor and plantarflexor) can be used to accurately predict noncyclic variations in amplitude and timing of ankle movement intrinsic to human walking across different terrains.

Unlike other nonlinear regressive neural networks (Zhang et al., 2012; Ardestani et al., 2014; Chen et al., 2018; Gupta et al., 2020), the current NARX model included temporal relationships (prediction interval, τ = *mΔt*) of inputs and outputs allowing for the prediction of future limb state. A crucial advantage of prediction (i.e., future estimates) for the control of active ankle-foot prostheses is the ability to counteract delays from prosthetic actuation, signal processing (e.g., filtering, sampling), and sensor response inherent to electromechanical systems. In this study, the performance of the optimized NARX models was evaluated using a prediction interval of 58 ms to account for microcontroller and motor actuation delays (max. 50 ms) inherent to Marquette University’s active powered ankle prosthesis (Sun and Voglewede, 2012; Sun et al., 2014; Klein and Voglewede, 2018). Other active ankle-foot designs have reported time delays of 40 ms for the system delay between the input and output of a two degrees of freedom cable-driven prosthesis (Ficanha et al., 2016), and maximum 40 ms for the pull-in response time of a bypass restriction valve of an electrohydrostatic-based prosthesis (Yu, 2017). Given the robust performance over a wide range of prediction intervals, sampling windows, and number of hidden units (Figure 4), models containing larger (or smaller) prediction intervals could be used with comparable results.

Despite variations in ankle moment, walking speed, and muscle activation patterns, the prediction error remained within the range of walking variability measured across ambulation conditions. The use of two antagonist muscles as inputs, mainly responsible for sagittal ankle motion, resulted in minimal output oscillations that can occur when redundant information is present across multiple inputs (Zhang et al., 2012; Ardestani et al., 2014; Gupta et al., 2020), and simplified the intrinsic (EMG) (Ardestani et al., 2014; Chen et al., 2018) and extrinsic (e.g., knee angle, hip angular velocity) (Ardestani et al., 2014; Dey et al., 2019; Eslamy and Alipour, 2019; Gupta et al., 2020) inputs needed for implementation in a myoelectric ankle-foot prosthesis.

The NARX model performance complements and extends other feedforward estimation approaches for continuously estimating ankle kinematics and kinetics in healthy individuals (Zhang et al., 2012; Chen et al., 2018; Dey et al., 2019; Eslamy and Alipour, 2019; Gupta et al., 2020; Keleş and Yucesoy, 2020) and impaired patients (Zhang et al., 2012; Ardestani et al., 2014; Farmer et al., 2014). Across studies in health individuals, reported errors (RMSE) and correlations (R^2^) of ankle angle varied between 2.44 and 5.29 degrees, and between 0.74 and 0.94, respectively, during level walking at different speeds and with various inputs. Specifically, Gupta et al. reported ankle angle errors, across healthy participants, during level walking (RMSE = 2.44 ± 0.45°, *r* = 0.97), stair ascent (RMSE = 3.61 ± 1.00°, *r* = 0.93), and stair descent (RMSE = 5.04 ± 1.56°, *r* = 0.85) using subject-specific NARX models trained and tested separately for each condition (Gupta et al., 2020). In comparison, average angle errors in this study were a factor of five lower for networks trained across the three terrains (RMSE < 0.84°). This may be tied to differences in the data organization (i.e., concurrent set of sequences) and the division of trials (i.e., contiguous blocks) used here during training which minimized discontinuities in the data that would cause inherent training errors, and ensured that random trials, instead of random points, were used during training. Ardestani et al. obtained ankle moment errors up to 8 and 13% using FFANN and WNN models, respectively, during normal walking in patients with unilateral knee replacement (Ardestani et al., 2014). Interestingly, their results were based on a non-specific inter-subject training paradigm wherein the network was trained on data from three different patients while performing three walking conditions, and tested on a fourth participant. In their study, the purpose was to create a generalized real-time surrogate inverse dynamic model for gait analysis and was not intended for use in prosthetic control. Dey et al. used an SVR approach to continuously estimate ankle angle and moment during level ground walking (RMSE = 2.17° and 0.11 Nm/kg, respectively) using two extrinsic inputs (hip and knee angle) of a single healthy participant (Dey et al., 2019). The use of kinematic-only inputs could provide an alternative for predicting user intent indirectly but the need for wearable sensors extrinsic to the prosthesis poses similar challenges to EMG-based systems. Keleş et al. performed a comprehensive analysis of muscle sites and their combinations for use as EMG inputs to estimate ankle angle and moment during walking in healthy participants (Keleş and Yucesoy, 2020). Using the same ankle plantarflexor and dorsiflexor muscles, they reported ankle angle and moment errors (RMSE) of 2.34 ± 0.15 degrees and 0.041 ± 0.006 Nm/kg, in comparison to 0.84 ± 0.23 degrees and 0.026 ± 0.006 Nm/kg in this study. Correlations were comparable to those reported here, although performance was not examined across ambulation conditions.

The robust performance of the feedforward NARX model across ambulation conditions and terrain transitions suggest that it could provide intuitive user-driven control of an active powered ankle-foot prosthesis, however, additional work is needed. Implementation in a physical system will require a closed-loop architecture wherein previous values of the predicted (rather than desired) ankle angle and moment are used to estimate changes in ankle dynamics. Feedback of the model predictions is expected to place greater emphasis on the use of EMG inputs to control for errors in the predicted ankle dynamics and to signal user intent during transitions and noncyclic activities (e.g., standing, sitting, obstacle avoidance). While previous studies have demonstrated the feasibility of continuously predicting ankle angle (Zhang et al., 2012; Farmer et al., 2014) and ankle moment (Ardestani et al., 2014), independently from neurological and neuromuscular impaired participants, the simultaneous prediction of ankle angle and moment across ambulation conditions must also be demonstrated with pathological muscle activity and gait data. Lastly, the effects on model performance associated with changes in EMG signal over time caused by variations in sensor placement and electrode-skin conductivity were not evaluated. Future work will characterize closed-loop NARX model performance across ambulation conditions using EMG activity from amputees’ residual lower limb muscles as inputs. Furthermore, for real-time implementation in a prosthetic design, the model would be validated with data acquired from commonly used angle and moment sensors [e.g., encoders, inertial measurement units, force sensitive resistors, potentiometers, torque, load cells (Au et al., 2007; Sup et al., 2009; Klein and Voglewede, 2018)] intrinsic to the prosthesis instead of motion capture and force plate data as done in this study.

## Conclusion

This study has demonstrated that a single-network nonlinear autoregressive model with exogenous EMG inputs can continuously predict future ankle angle and ankle moment simultaneously during normal walking across ambulation conditions (level ground walking, stair ascent/descent) and transitions between terrains. The natural patterns of muscle activation used to predict variations in normal gait, particularly during transitions, suggests that this approach could be used to create a seamless and intuitive interface for an active powered ankle-foot prosthesis that incorporates user intent and does not require conscious user control. The model’s accuracy, robustness, and predictive capabilities (i.e., future estimates) suggest that the approach could be adapted for real-time closed-loop control of a wide variety of lower limb robotic devices, including actuated orthoses, and exoskeletons. Further research will characterize the ability of within-socket residual EMG activity from amputees to continuously predict limb kinematics and kinetics across a variety of ambulation conditions.

## Data Availability Statement

The raw data supporting the conclusions of this article will be made available by the authors, without undue reservation.

## Ethics Statement

The studies involving human participants were reviewed and approved by Marquette University Institutional Review Board. The patients/participants provided their written informed consent to participate in this study.

## Author Contributions

EZ-G contributed to conception and design of the study, performed acquisition, analysis and interpretation of data, and drafted the manuscript. LR supported data acquisition and processed motion data. PV, BS-T, SK-M, and SB contributed to conception and design of the study, obtained funding, and supervised the study. All authors contributed to manuscript revision and approved the submitted version.

## Conflict of Interest

The authors declare that the research was conducted in the absence of any commercial or financial relationships that could be construed as a potential conflict of interest.

## Publisher’s Note

All claims expressed in this article are solely those of the authors and do not necessarily represent those of their affiliated organizations, or those of the publisher, the editors and the reviewers. Any product that may be evaluated in this article, or claim that may be made by its manufacturer, is not guaranteed or endorsed by the publisher.
