# Supplementary material for: Continuous Myoelectric Prediction of Future Ankle Angle and Moment Across Ambulation Conditions and Their Transitions
Source: Front Neurosci. 2021 Aug 18;15:709422. doi: 10.3389/fnins.2021.709422 (PMC8416349; doi:10.3389/fnins.2021.709422)
Supplement: Supplementary file 1 [file Data_Sheet_1.pdf]

Supplementary Table 1. Model predictions and targets of ankle angle and ankle moment at critical performance points across participants for each ambulation condition. Significant differences between experimentally measured targets and the model predictions were assessed using a paired-samples t-test (normally distributed samples) and Sign test (non-normally and asymmetric distributed samples). Bold numbers indicate significance using the Benjamini-Hochberg (B-H) multiple comparisons procedure with a false discovery rate of 0.05. Shaded areas represent staircase transition gait cycles during stair ambulation.

**Critical Performance Points**	**Units**	**Mean ± SD**		**t (df)**	***p* value**	**B-H *p* value**	**Mean ± SD**		**t (df)**	***p* value**	**B-H *p* value**	**Mean ± SD**		**t (df)**	***p* value**	**B-H *p* value**
		**Target**	**Prediction**				**Target**	**Prediction**				**Target**	**Prediction**			
							**Level Ground (n=10)**									
							**Gait Cycle 1**									
Clearance Intervals during swing phase																
Interval 1: MTC (Mean - SD)	Degrees						-6.03 ± 3.93	-6.03 ± 3.72	t(9)= -0.010	0.992	*0.992*					
Interval 1: MTC (Mean)	Degrees						-3.56 ± 3.73	-3.67 ± 3.79	t(9)= 1.507	0.166	*0.407*					
Interval 1: MTC (Mean - SD)	Degrees						-1.04 ± 3.55	-0.92 ± 3.56	t(9)= -1.463	0.178	*0.414*					
Interval 2: MFC (Mean + SD)	Degrees						2.44 ± 3.15	2.34 ±3.10	t(9)= 1.203	0.260	*0.471*					
Interval 2: MFC (Mean)	Degrees						3.33 ± 2.89	3.09 ± 2.66	Sign Test	0.344	*0.581*					
Interval 2: MFC (Mean + SD)	Degrees						3.80 ± 3.09	3.53 ± 2.74	t(9)= 2.163	0.059	*0.213*					
Stance Phase Critical Points																
Flexion at Toe Off	Degrees						-2.00 ± 2.10	-1.97 ± 1.94	t(9)= -0.186	0.857	*0.893*					
Dorsiflexion	Degrees						13.93 ± 1.94	13.99 ± 1.88	t(9)= -1.303	0.225	*0.455*					
Plantarflexion	Degrees						-4.56 ± 4.59	-4.77 ± 4.76	t(9)= 1.518	0.163	*0.407*					
Plantarflexor moment	Nm/kg						1.64 ± 0.11	1.62 ± 0.10	t(9)= 4.577	0.001	*0.065*					
							**Stair Ascent (n=8)**									
		**Gait Cycle 1**					**Gait Cycle 2**					**Gait Cycle 3**				
Clearance Intervals during swing phase																
Interval 1: MTC (Mean - SD)	Degrees	12.35 ± 4.77	12.20 ± 4.77	t(7)= 2.825	0.026	*0.125*	11.57 ± 6.19	11.60 ± 6.25	t(7)= -0.301	0.772	*0.823* [b]	a				
Interval 1: MTC (Mean)	Degrees	13.70 ± 4.35	13.55 ± 4.45	t(7)= 1.408	0.202	*0.450* [b]	13.22 ± 5.78	13.34 ± 5.82	t(7)= -1.349	0.219	*0.455* [b]	a				
Interval 1: MTC (Mean - SD)	Degrees	14.37 ± 4.12	14.33 ± 3.97	t(7)= 0.348	0.738	*0.803* [b]	14.73 ± 5.29	14.78 ± 5.39	Sign Test	0.727	*0.803* [b]	a				
Interval 2: MFC (Mean + SD)	Degrees	13.70 ± 4.35	13.55 ± 4.45	t(7)= 1.408	0.202	*0.450* [b]	-8.61 ± 5.69	-8.84 ± 5.56	t(7)= 2.231	0.061	*0.213*	-7.98 ± 5.01	-8.05 ± 4.85	t(7)= 0.584	0.578	*0.765* [c]
Interval 2: MFC (Mean)	Degrees	14.37 ± 4.12	14.33 ± 3.97	t(7)= 0.348	0.738	*0.803* [b]	-15.03 ± 4.70	-15.75 ± 4.52	t(7)= 3.551	0.009	*0.065*	-15.06 ± 4.56	-14.90 ± 4.41	t(7)= -0.902	0.397	*0.600*
Interval 2: MFC (Mean + SD)	Degrees	14.15 ± 4.18	14.02 ± 4.24	t(7)= 1.247	0.252	*0.471*	-9.83 ± 5.81	-9.91 ± 5.76	t(7)= 0.413	0.692	*0.803*	-14.98 ± 5.27	-14.85 ± 5.43	t(7)= -0.887	0.404	*0.600*
Stance Phase Critical Points																
Flexion at Toe Off	Degrees	6.17 ± 2.77	6.37 ± 2.58	Sign Test	0.727	*0.803*	-6.95 ± 5.64	-7.12 ± 5.43	t(7)= 1.683	0.136	*0.393* [c]	-7.98 ± 5.01	-8.05 ± 4.85	t(7)= 0.584	0.578	*0.765* [c]
Dorsiflexion	Degrees	d					16.10 ± 3.00	16.04 ± 3.00	t(7)= 1.078	0.317	*0.555*	d				
Plantarflexion	Degrees	d					-6.95 ± 5.64	-7.12 ± 5.43	t(7)= 1.683	0.136	*0.393* [c]	d				
Plantarflexor moment	Nm/kg	1.58 ± 0.11	1.58 ± 0.12	t(7)= 0.616	0.557	*0.759*	1.59 ± 0.20	1.59 ± 0.21	t(7)= -0.674	0.522	*0.730*	1.55 ± 0.21	1.55 ± 0.20	t(7)= -0.975	0.362	*0.591*
							**Stair Descent (n=8)**									
		**Gait Cycle 1**					**Gait Cycle 2**					**Gait Cycle 3**				
Clearance Intervals during swing phase																
Interval 1: MTC (Mean - SD)	Degrees	9.03 ± 5.05	9.09 ± 4.97	t(7)= -0.812	0.444	*0.639*	10.37 ± 4.29	10.20 ± 4.44	t(7)= 2.090	0.075	*0.245*	a				
Interval 1: MTC (Mean)	Degrees	6.82 ± 4.79	7.04 ± 4.79	t(7)= -3.574	0.009	*0.065*	9.29 ± 3.98	9.20 ± 4.07	t(7)= 0.935	0.381	*0.600*	a				
Interval 1: MTC (Mean - SD)	Degrees	2.89 ± 4.89	3.26 ± 5.06	t(7)= -4.502	0.003	*0.065*	7.45 ± 3.64	7.44 ± 3.66	t(7)= 0.162	0.876	*0.894*	a				
Interval 2: MFC (Mean + SD)	Degrees	-4.99 ± 5.91	-4.54 ± 6.05	t(7)= -3.391	0.012	*0.068*	5.26 ± 3.75	5.33 ± 3.73	t(7)= -0.487	0.641	*0.786*	a				
Interval 2: MFC (Mean)	Degrees	-8.53 ± 6.17	-8.13 ± 6.23	t(7)= -3.706	0.008	*0.065*	2.15 ± 4.46	2.33 ± 4.41	t(7)= -1.308	0.232	*0.455*	a				
Interval 2: MFC (Mean + SD)	Degrees	-10.96 ± 6.28	-10.61 ± 6.39	t(7)= -3.339	0.012	*0.068*	-2.06 ± 5.65	-1.75 ± 5.64	t(7)= -1.834	0.109	*0.335*	a				
Stance Phase Critical Points																
Flexion at Toe Off	Degrees	19.89 ± 4.47	19.94 ± 4.45	t(7)= -0.506	0.629	*0.786*	21.14 ± 3.83	21.25 ± 3.88	t(7)= -1.606	0.152	*0.407*	-1.37 ± 4.22	-1.05 ± 4.23	t(7)= -3.722	0.007	*0.065*
Dorsiflexion	Degrees	d					32.94 ± 4.05	32.79 ± 4.10	t(7)= 2.644	0.033	*0.148*	d				
Plantarflexion	Degrees	d					-23.01 ± 1.88	-22.82 ± 1.77	t(7)= -2.487	0.042	*0.170*	d				
Plantarflexor moment	Nm/kg	1.24 ± 0.21	1.24 ± 0.21	t(7)= -0.550	0.600	*0.773*	1.20 ± 0.23	1.22 ± 0.22	t(7)= -4.035	0.005	*0.065*	1.71 ± 0.17	1.70 ± 0.18	t(7)= 0.382	0.714	*0.803*

MTC, Minimum Toe Clearance; MFC, Minimum Foot Clearance; SD, Standard Deviation

[a] No MTC or MFC was reported for an equivalent step in literature reference

[b] Overlapped MTC and MFC intervals

[c] Point overlaps with another critical point

[d] Only stair step analyzed
